# Individual- and institution-level predictors of the turnover intention of medical staff among rural primary medical institutions in Xinjiang Production and Construction Corps, China: a cross-sectional multi-level analysis

**DOI:** 10.3389/fpsyg.2023.1112057

**Published:** 2023-08-10

**Authors:** Taoyu Lin, Ye Li, Yuanyuan Li, Wei Guo, Xiaoying Guo, Changmin Tang

**Affiliations:** ^1^The People’s Hospital of Suzhou New District, Suzhou, Jiangsu, China; ^2^School of Medicine, Shihezi University, Shihezi, Xinjiang, China; ^3^School of Management, Hubei University of Chinese Medicine, Wuhan, Hubei, China

**Keywords:** primary medical staff, turnover intention, multi-level analysis, rural primary medical institution, China

## Abstract

**Background:**

Primary medical staff (PMS) are the guardians of population health. However, their loss further worsens the shortage and uneven distribution of human health resources, which should be addressed immediately. This study aimed to investigate the current status of turnover intention of rural PMS in Xinjiang Production and Construction Corps (XPCC) in China and its influencing factors atthe individual and institutional levels to provide reliable baseline data for intervention strategies to protect valuable rural PMS.

**Methods:**

Participants were recruited from rural public health institutions of the XPCC using a cross-sectional multistage sampling process. Data on participants’ turnover intention and individual- and institution-level indicators were obtained through standardized electronic questionnaires and statistical reports of regional health administrative departments. The key factors influencing PMS turnover intention were identified us ingunivariateandmulti-level logistic regression analysis.

**Findings:**

Overall, 20.5% (447/2182) of participants reported turnover intention. Univariate analysis showed that the occurrence of turnover intention was significantly influenced by marriage, education, age, year of working, monthly income, human resource management practices (HRMP), job satisfaction, *per capita* served population (PCSP) and number of beds (*p* < 0.05). Multi-level logistic regression analysis showed that bachelor’s degree or above and intermediate professional title were closely related to the occurrence of turnover intention (*p* < 0.05), age 41–50 years old and above, high human resource management practice, and high job satisfaction effectively reduced the odds (*p* < 0.05). The odds of turnover intention increased by 37% (*p* < 0.10) for PMS in institutions with PCSP more than 250 people. In contrast, the odds of turnover intention decreased to 68% (*p* < 0.05) for PMS in institutions with more than 50 beds.

**Conclusion:**

Government-run primary medical institutions face the risk of PMS turnover intention. From a personal perspective, the high-risk population fortheturnover intention was mainly the PMS with bachelor’s degrees or above and intermediate professional titles. The low-risk population was the PMS with aged over 40 years, a higher evaluation of human resource management practice, and job satisfaction. From the perspective of primary medical institutions, larger institutions can reduce the turnover intention of individuals, whereas the size of the service population has the opposite effect.

## 1. Introduction

Primary medical staff (PMS) are guardians of population health. Appropriate PMS plays a vital role in controlling costs, promoting rational utilization of healthcare services, and improving equity in population access to healthcare services (Kuhlmann et al., 2018). Many countries have long been committed to improvingPMS allocation and spatial distribution (World Health Organization, 2022a). However, with the aging of the population and the change in the disease spectrum, shortage and uneven distribution of PMS remain widespread, and the loss of PMS has always been an urgent problem to be overcome (World Health Organization, 2017; Skinner et al., 2019; World Health Organization, 2022a).

**Figure 1:**
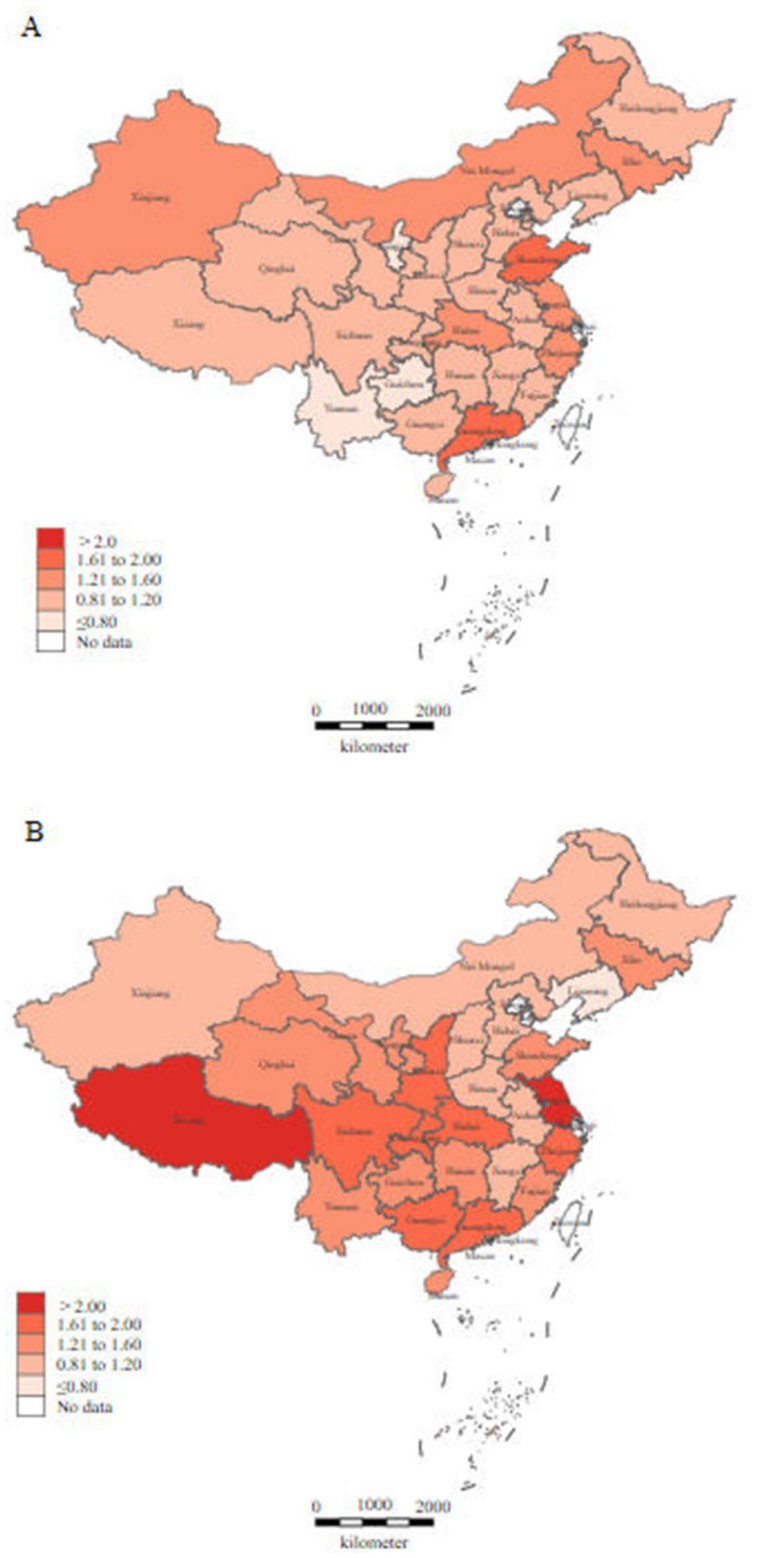
Geographical distribution of rural PMS density in 2010 **(A)** and 2020 **(B)**. The data were collected from China Health Statistical Yearbook 2011 and China Health Statistical Yearbook 2021.

**Figure 2:**
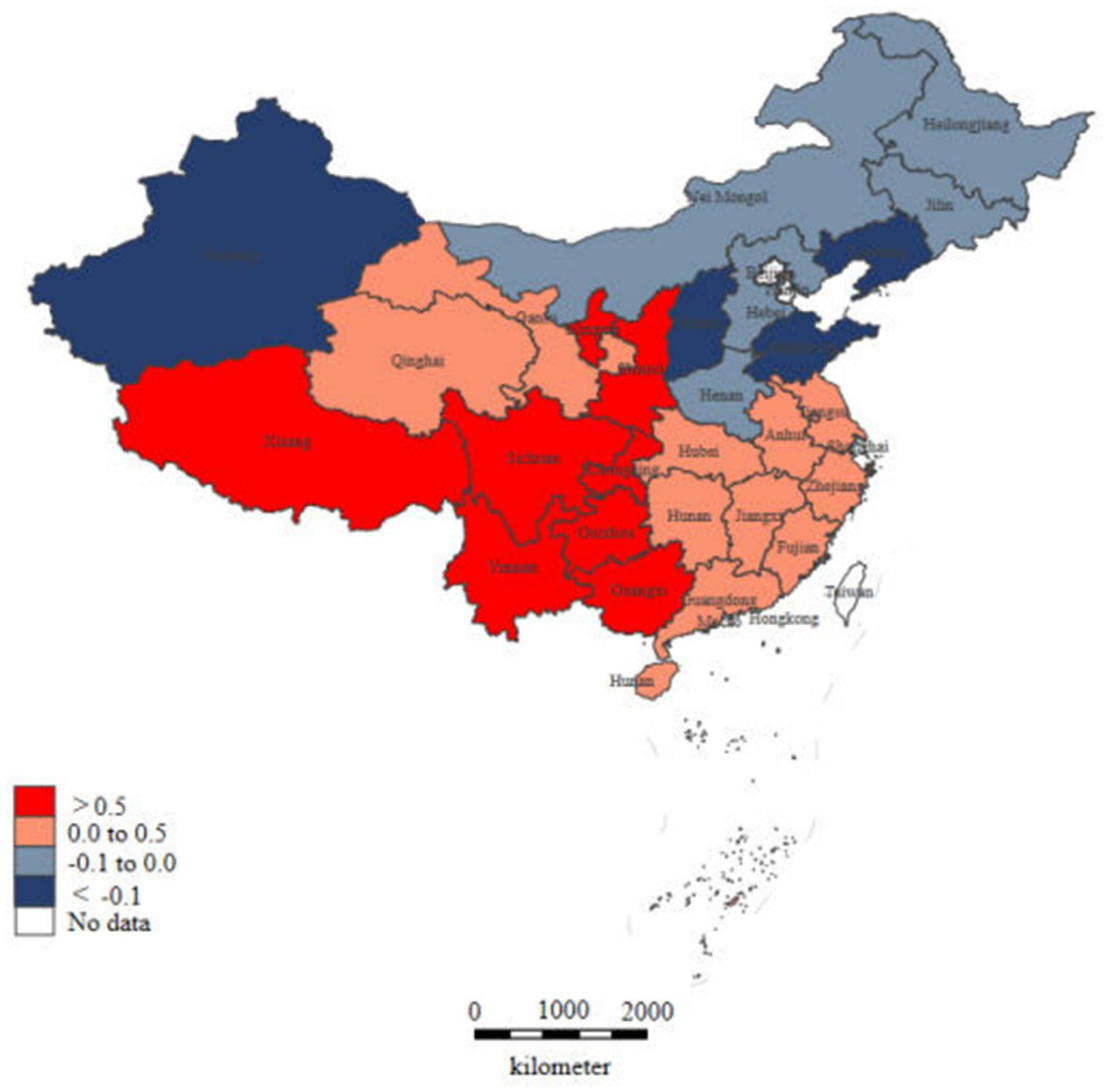
Changes in rural PMS density between 2010 and 2020. The data were collected from China Health Statistical Yearbook 2011 and China Health Statistical Yearbook 2021.

China’s aging population is large and growing rapidly. Compared with highly aging countries, such as France, Great Britain, Australia, and Japan, the Chinese population has a low healthy life expectancy (1.6–2.6 years lower) and fewer doctors providing health services (2.5–19.0 fewer doctors per 1,000 population) (World Health Organization, 2022b). Notably, rural areas were even more worried. The population aged 65 years and older (17.7%) was 5.3% higher than the national average, and 60% of them had chronic diseases (National Bureau of Statistics, 2021; National Health Commission Statistical Information Center, 2021). Although the total number ofPMSs providing basic medical services to rural residents increased, the equity of their distribution showed a downward trend (Figure 1). In the four provinces with the most severe decrease in PMS density (Figure 2), Xinjiang’s vast territory (accounting for 1/6 of the national geographic area), and sparse population (only 1.83% of the national population), the regional distribution of rural PMS is more unfair than that of Liaoning, Shandong, and Shanxi provinces.

The Xinjiang Production and Construction Corps (XPCC), which is a special organization planned separately by China, is an important part of Xinjiang. Meanwhile, it is one of the core regions of China’s “silk road economic belt” and the maintenance of national stability.Its geographical area covers almost the entire territory of Xinjiang, making the sparse population (less than 13% of Xinjiang’s total population) more dependent on the services provided by the PMS (Li and Yu, 2020; Liu and Ye, 2020). However, the high aggregation of medical staff in urban areas has further altered the density of rural PMS (PMS density was lower than the national average) and caused the loss of PMS (Lei et al., 2014). To date, little is known about this phenomenon. Therefore, predicting the loss of PMS in XPCC and tracing the root causes arecrucial for the sustainable development of PMS and the health level ofpopulation in this region, as well asthe socio-economic development in China.

Turnover intention is the direct antecedent variable of turnover behavior (Al-Dabbagh et al., 2022), which is easier to measure and more accurate than the turnover information provided by those who have already resigned (de Oliveira et al., 2019). In China, 11.8–78.4% of PMS were strongly willingto leave (Mao et al., 2018). Compared with urban PMS, the risk of leaving rural PMSwas higher by 1.3 times (Mao et al., 2018). According to a survey, 43.7% of PMS with turnover intention wanted to leave the health profession (Liu et al., 2017), and among those who left,9.8% had switched to other industries (Li et al., 2021).

Evidence has established that the strength of turnover intention is influenced by individual factors, including age, marriage, education, seniority, wages, benefits, and subjective attitude (Liu and Mao, 2016; Liu et al., 2017; Kao et al., 2018; Ou et al., 2018; Liu et al., 2019; Worku et al., 2019; Bardoel et al., 2020; Gan et al., 2020; Li et al., 2020; Ran et al., 2020; Anderson et al., 2021; Kong et al., 2021; Li et al., 2021; Deng et al., 2022). Several factors at the organizational level also directly or indirectly affect the turnover intention of individuals, including organizational culture, commitment, empowermentand poor working conditions (Sun et al., 2013; Lee et al., 2016; Eneroth et al., 2017; Zhang et al., 2017; Kao et al., 2018; Worku et al., 2019; Anderson et al., 2021; Li et al., 2021; Hwang and Yi, 2022), and their effects may be stronger than those of individual factors (Anderson et al., 2021; Ahmed et al., 2022).Under the background of the equalization policy of basic public health services, an increase in service radius and service population of an institutionfurther aggravates the work burden of PMS, which is more likely to lead to strong turnover intention of individuals (Anderson et al., 2021; Li et al., 2021).

However, due to the inconsistency of study areas and participants, there was large heterogeneity in the research results. First, the groups at high riskof turnover intention differed. For example, a national study (Zhang et al., 2017) and another study from Shaanxi (Liu and Mao, 2016) had different findings on the high-risk age of turnover intention of PMS (31–45 vs. ≤30 years). Second, human resource management practice (HRMP) is key for preventing medical staff turnover and maintainingsustainable development (Onnis, 2019). However, previous studies mostly explored managers’ perspectives (He et al., 2020, 2022) but ignored individual perceptions. Finally, individual turnover intentions are aggregated at different levels (e.g., organizations and regions). However, the existing literature mainly analyzes at the individual level (Gan et al., 2020; Lee and Jang, 2020; Deng et al., 2022) and pays little attention to the differences between primary medical institutions. Therefore, a comprehensive analysis of the odds of PMS turnover intentionatindividual and organizational levels is necessary.

From a long-term development perspective, the key challenge facing the XPCC is to systematically trace the root of rural PMS turnover intention at both the institutional and individual levels and effectively improve rural residents’ access to basic medical services. Therefore, this study selected the rural PMS of the XPCC as the sample, adopted multi-level mixed effect models to capture the multiple hierarchical data structures of turnover intention, predicted the turnover intention of rural PMS, and determined the key influencing factors at the individual and institutional levels, to provide reliable baseline data for intervention strategies to protect valuable rural PMS and to provide a reference for similar regions and countries.

## 2. Materials and methods

### 2.1. Study design and data sources

**Figure 3:**
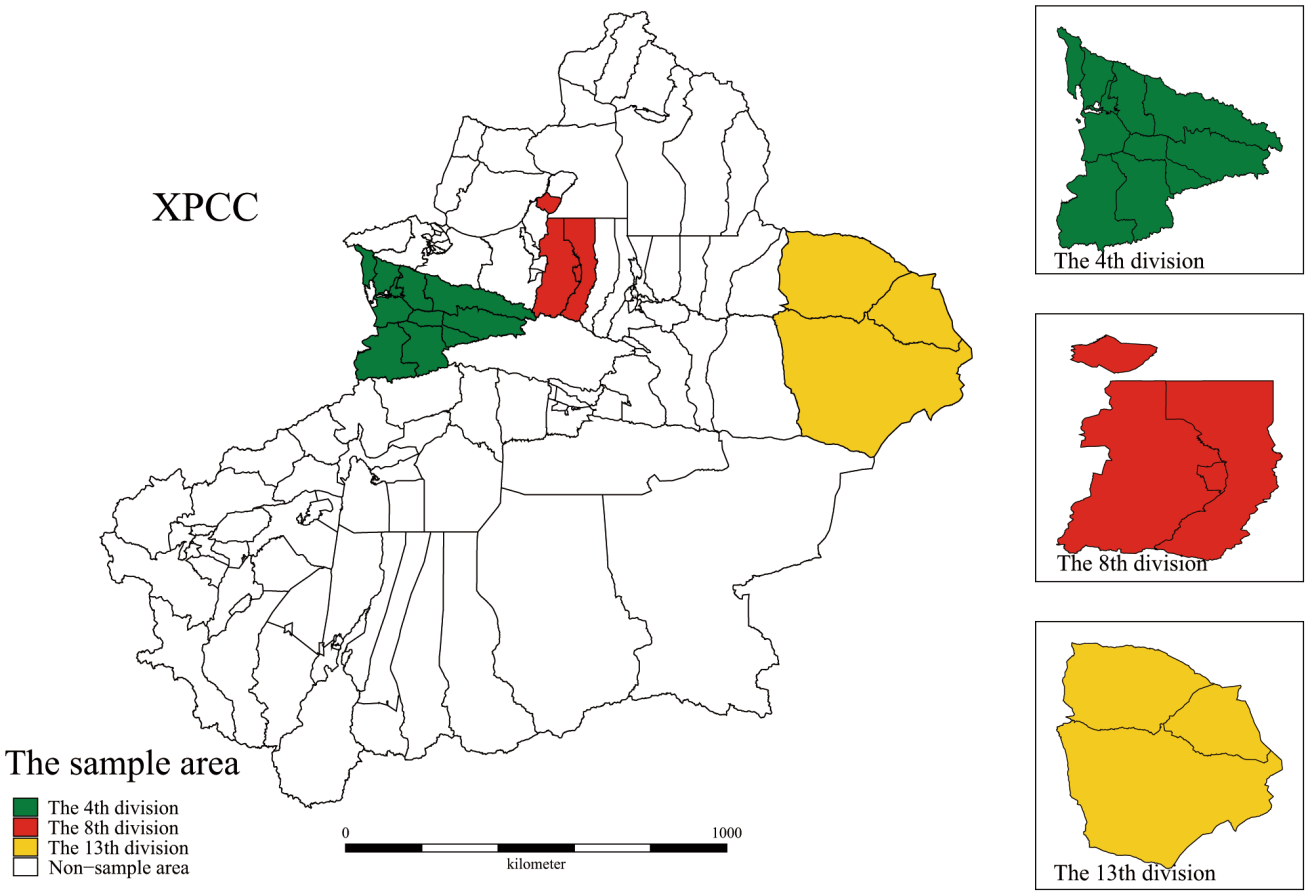
Geographic location of the sampling area.

In this cross-sectional survey design, participants were selected from rural public primary medical institutions in the XPCC using stratified multistage sampling methods.The sampling procedure was categorized into the following four stages. In Stage 1, according to the population size at the end of 2020, 14 divisions of the XPCC were categorized into high population size (ranking the top 4 in population size), medium population size (ranking 5–9 in population size), and small population size (ranking 10–14 in population size). One division on each layer was randomly selected as the sample division in Stage 2 (Figure 3). In Stage 3, all rural public primary medical institutions in each sample division were selected using cluster sampling. Finally, PMS were randomly selected from selected primary public health institutions and stratified by occupation and professional title. Furthermore, participants were screened at an individual level, and eligible individuals were doctors, nurses, and other medical technicians working for 1or more years. Those who refused to participate orcouldnotcomplete the questionnaire independently were excluded.

Measurement indicators and tools were determined based on previous studies and thematic panel discussions. The indicator data for this study were obtained from two sources: demographic characteristics, personal perception of the organizational environment, and turnover intention were obtained from the standardized electronic questionnaire of the participants; data reflecting the institutional resource allocation were obtained from the statistical statements of the health administrative departments of the sample areas.

### 2.2. Measure

After obtaining written informed consent, all eligible participants independently completed a standardized electronic questionnaire. The questionnaire included basic information about the participants, HRMP, job satisfaction, and turnover intention.This study used measurement tools effectively applied in previous studies to measure HRMP, job satisfaction, and turnover intention to ensure the validity and accuracy of the measurement. All items were scored on a5-point Likert scale ranging from 1 (strongly disagree) to 5(strongly agree). When the average score of each scale was more than 3 points, it was counted as 1 point (indicating that the indicators of turnover intention, HRMP, and job satisfaction were at a high level), and the rest were 0 points (indicating that the indicators above were at a low level). Furthermore, the number of beds, health technicians in primary medical institutions, and population served by primary medical institutions (including the age structure of the population) in 2020 were extracted from the data in the statistical statements, which the related person in charge of the health administration of the sample region provided. In all surveys, quality-assurance procedures were incorporated to improve the data quality.

### 2.3. Outcome indicator

The turnover intention was measured using a 5-item scale (1 = “strongly disagree” to 5 = “strongly agree”) developed by Mobley (1977) and adapted by Lei et al. (2020) with the working status of medical staff in China. The higher the score, the stronger the turnover intention. The reliability and validity of the scale have been validated in previous studies (Cronbach’s alpha was 0.9, the KaiserMeyerOlkin (KMO) value was 0.8, and the single-factorial model explained 82.8% of the overall variance) (Lei et al., 2020).

### 2.4. Individual-level indicators

Individual-level indicators mainly consisted of two components: the participants’personal characteristics and their perceptions of HRMP and job satisfaction. The former includes gender (female and male), age (≤30, 31–40, 41–50, and ≥ 51 years), marital status (married and single), education level (technical secondary school, junior college, bachelor’s degree, and above), occupation (doctor, nurse, and other medical staff), professional title (junior title, middle title, senior professional title), working years (≤10, 11–20, and ≥ 21 years), and monthly income (≤4,000 yuan and > 4,000 yuan). The latter was measured using the following scales:

The HRMP measure was based on a 15-item scale adapted by Lan (2012), which comprises three subscales: selection and allocation (three items), training and development (five items), performance, and salary management (seven items). The Cronbach’s alpha of the scale was 0.9, and the KMO value was >0.6. Three common factors were extracted through exploratory factor analysis, and the cumulative contribution rate was >70% (Lan, 2012; He et al., 2020).

Job satisfaction was measured using the Minnesota Satisfaction Scale (Weiss et al., 1967), with a total of 20 items consisting of two subscales of satisfaction and external satisfaction. This scale has been widely used to measure the job satisfaction of medical staff in China and has good validity and reliability (Cronbach’s *a* = 0.87–0.92, KMO >0.6; the cumulative contribution rate of the two common factors extracted was 64.2%) (Lan, 2012).

### 2.5. Institutional-level indicators

Institutional-level indicators in this study included two indicators: (1) the number of beds in primary medical institutions was a categorical variable (≤50 and > 50 beds) and (2) *per capita* served population (PCSP) was defined as the average number of the population served per PMS. The calculation formula is as follows:

PCSP = population served by the primary medical institution/total number of PMS in primary medical institutions.

Because the optimal number of people served per PMS was unknown, the PCSP was classified into two categories: 250 or fewer people serviced per PMS and more than 250 people serviced per PMS.

### 2.6. Statistical analysis

All statistical analyses were performed using the STATA 16.1(Stata Corporation, College Station, TX, United States). First, the sample was analyzed using descriptive analysis, and the results were expressed as the frequency and proportion of categorical variables. Second, a univariate logistic regression model of turnover intention was constructed to screen for factors significantly related to turnover intention (*p* < 0.10). Third, a two-level logic model of PMS turnover intention was constructed, namely, level 1 (individual-level factors) and level 2 (institutional-level factors), which evaluated the determinants of turnover intention. Before performing the above processes, a null model with only a constant/intercept was used to verify the model’s validity for the dependent variables. The second model included individual-level indicators that significantly impactedPMS turnover intention. The final model included individual- and institutional-level indicators that significantly impactedPMS turnover intention. Significant results for all models are presented asodds ratios (ORs) with 95% confidence intervals (CI). The optimality criteria, including the Akaike Information Criterion (AIC) and log-likelihood ratio, were used for model comparison. The model with the smallest AIC was judged to have the best fit (Zhang et al., 2022). All tests were two-tailed, and a significant difference was set at *p* < 0.05.

## 3. Results

### 3.1. Descriptive analysis

As presentedin Table 1, 2,182 PMS from 44 primary medical institutions were surveyed. Most PMS were female, aged 41–50 years old, married, junior college, doctorate, junior title, working time of more than 20 years, and had a monthly income of more than 4,000 yuan. Regarding personal perceptionsatthe organizational level, approximately 67.7% of PMS made high evaluations of the institution’s HRMP (rate 4 or 5), and approximately 55.7% of PMS reported that they were satisfied with their jobs (rate 4 or 5). Regarding institutional-level indicators, 55.7% of PMSs worked in primary medical institutions with more than 50 beds, and 88.3% of PMSs worked in institutions with a PCSP of no more than 250 people. Overall, 20.5% (447/2182) of participants reported turnover intention. The distribution of this sample issummarized in Table 1.

**Table 1:** Descriptive statistics of individual level indicators and institution level indicators of the PMS.

**Demographic variable**	Total (*N* = 2,182)	Turnover intention	
		Yes (*n* = 447)	No (*n* = 1735)
**Individual-level indicators**			
*Gender*			
Male	504 (23.1)	116 (26.0)	388 (22.4)
Female	1,678 (76.9)	331 (74.0)	1,347 (77.6)
*Age (year)*			
≤30	431 (19.8)	130 (29.1)	301 (17.4)
31–40	280 (12.8)	86 (19.2)	194 (11.2)
41–50	896 (41.1)	161 (36.0)	735 (42.4)
>50	575 (26.4)	70 (15.7)	505 (29.1)
*Marital status*			
Married	1831 (83.9)	358 (80.1)	1,473 (80.4)
Single	351 (16.1)	89 (19.9)	262 (19.6)
*Education level*			
Technical secondary school	431 (19.8)	54 (12.1)	377 (21.7)
Junior college	1,352 (62.0)	250 (55.9)	1,102 (63.5)
Bachelor degree and above	399 (18.3)	143 (32.0)	256 (14.8)
*Occupation*			
Doctor	1,187 (54.4)	255 (57.1)	932 (53.7)
Nurse	954 (43.7)	182 (40.7)	772 (44.5)
Other medical staff	41 (1.9)	10 (2.2)	31 (1.8)
*Professional titles*			
Junior title	1,227 (56.2)	264 (59.1)	963 (55.5)
Middle title	614 (28.1)	126 (28.2)	488 (28.1)
Senior professional title	341 (15.6)	57 (12.8)	284 (16.4)
*Years of working (year)*			
≤10	232 (10.6)	68 (15.2)	164 (9.5)
11–20	535 (24.5)	108 (24.2)	427 (24.6)
>20	1,415 (64.9)	271 (60.6)	1,144 (65.9)
*Monthly income (yuan)*			
≤4,000	1,069 (49.0)	261 (58.4)	808 (46.6)
>4,000	1,113 (51.0)	186 (41.6)	927 (53.4)
*HRMP*			
Low	706 (32.4)	292 (65.3)	414 (23.9)
High	1,476 (67.6)	155 (34.7)	1,321 (76.1)
*Job satisfaction*			
Low	1,216 (55.7)	378 (84.6)	838 (48.3)
High	966 (44.3)	69 (15.4)	897 (51.7)
**Institutional-level indicators**			
*Bed number (beds)*			
≤50	966 (44.3)	242 (54.1)	724 (41.7)
>50	1,216 (55.7)	205 (45.9)	1,011 (58.3)
*PCSP (people)*			
≤250	1927 (88.3)	374 (83.7)	1,553 (89.5)
>250	255 (11.7)	73 (16.3)	182 (10.5)

HRMP, human resource management practice; PCSP, Per capita service population. Numbers are *n* (%).

### 3.2. Univariate analysis

**Figure 4:**
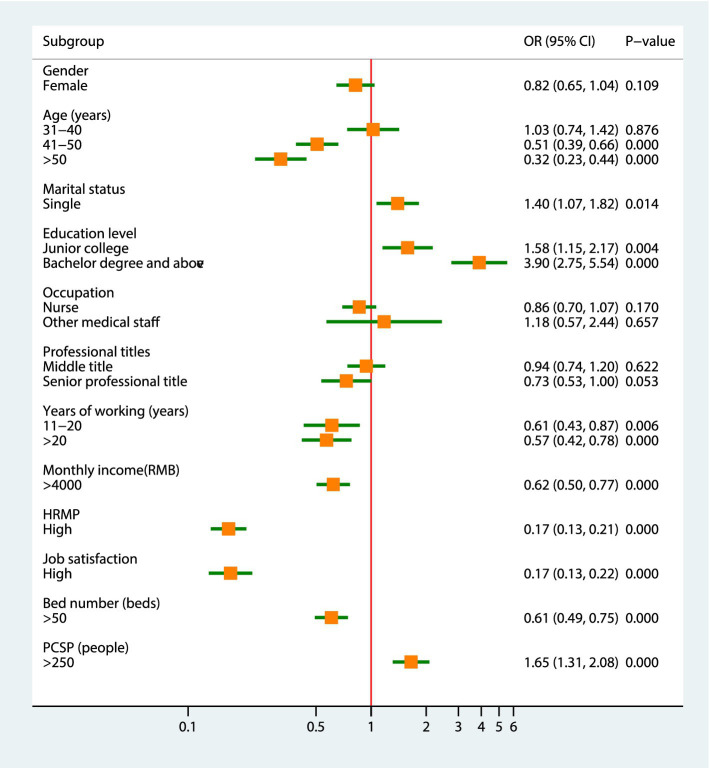
The results of the univariate analyses for PMS turnover intention based on individual- and institution-level indicators.

Figure 4 shows the results of the univariate analyses for PMS turnover intention based on individual- and institution-level indicators. Among the individual-level indicators, single and higher education levels were positively associated with turnover intention (*p* < 0.05 for all) and aged 41–50 years old and over 50 years, working time of more than 10 years, monthly income of more than 4,000 yuan, high HRMP level, and high job satisfactionwere negativelyassociated with turnover intention (*p* < 0.05 for all). Regarding the institutional-level indicators, the odds of turnover intention was significantly higher in PMS working at institutions with PCSP more than 250 people (OR: 1.65, 95%CI: 1.31–2.08, *p* < 0.001) than in those working at institutions with 250 people or less. In contrast, the odds of turnover intention was significantly lower in PMS working at institutions with more than 50 beds (OR: 0.61, 95%CI: 0.49–0.75, *p* < 0.001) than in those working at institutions with 50 beds or less.

### 3.3. Multi-level analysis

Three models were adapted to the multi-level logistic regression model (Table 2). The result of the random effects showed that in Model 1, which reflected the null model, the size of the intra-class correlation coefficient (ICC) was calculated to be 0.081, indicating that 8.1% of the variance in turnover intention is attributable to the institutional level (Kwon, 2020). The ICC values decreased to 4.4% in Model 2 (the inclusion of individual-level indicators that significantly influenced turnover intention in univariate analysis) and 2.3% in Model 3 (the full model with all the independent variables that significantly influenced turnover intention was considered). Compared with Model 1 and Model 2, Model 3 had the lowest Akaike information criterion (1770.51 *vs* 2167.88, 1771.17) and the highest log-likelihood ratio (−868.25 *vs* − 1081.94, −872.58). Therefore, Model 3 was considered to be the line of best fit suitable for predicting turnover intention among PMS.

**Table 2:** Mixed effects results on individual-, institutional-level indicators associated with PMS turnover intention.

Variable	Model 1 OR (95% CI)	Model 2 OR (95% CI)	Model 3 OR (95% CI)
**Individual variables**			
*Age (year)*			
≤30		1 (Ref)	1 (Ref)
31–40		0.72 (0.47–1.10)	0.73 (0.47–1.11)
41–50		0.32 (0.21–0.49)^***^	0.33 (0.21–0.51)^***^
>50		0.22 (0.14–0.37)^***^	0.23 (0.14–0.38)^***^
*Marital status*			
Married		1 (Ref)	1 (Ref)
Single		1.09 (0.75–1.57)	1.08 (0.75–1.56)
*Education level*			
Technical secondary school		1 (Ref)	1 (Ref)
Junior college		1.23 (0.86–1.77)	1.21 (0.85–1.74)
Bachelor degree and above		2.47 (1.60–3.83)^***^	2.40 (1.56–3.71)^***^
*Professional titles*			
Junior title		1 (Ref)	1 (Ref)
Middle title		1.49 (1.06–2.10) ^**^	1.51 (1.07–2.12)^**^
Senior professional title		1.33 (0.85–2.09)	1.37(0.88–2.14)
*Years of working (year)*			
≤10		1 (Ref)	1 (Ref)
11–20		1.13 (0.71–1.78)	1.13 (0.71–1.78)
≥21		1.09 (0.72–1.65)	1.09 (0.72–1.65)
*Monthly income (yuan)*			
≤4,000		1 (Ref)	1 (Ref)
>4,000		0.83 (0.61–1.14)	0.80 (0.59–1.10)
*HRMP*			
Low		1 (Ref)	1 (Ref)
High		0.25 (0.19–0.33)^***^	0.26 (0.20–0.34)^***^
*Job satisfaction*			
Low		1 (Ref)	1 (Ref)
High		0.30 (0.21–0.41)^***^	0.30 (0.21–0.41)^***^
**Institution variables**			
*Number of beds (beds)*			
≤50			1 (Ref)
>50			0.68 (0.50–0.92)^**^
*PCSP (people)*			
≤250			1 (Ref)
>250			1.37 (0.97–1.93) ^*^
*Random effect result*			
Variance (95% CI)	0.29 (0.15–0.55)	0.15 (0.06–0.40)	0.08 (0.02–0.35)
ICC	0.081	0.044	0.023
LR Test	48.95^***^	11.35^***^	3.00^*^
Wald chi-square	Reference	313.07^***^	323.87^***^
*Model fitness*			
Log-likelihood	−1081.94	−872.58	−868.25
AIC	2167.88	1771.17	1770.51
N	2,182	2,182	2,182

Model 1is the null model, a baseline model without any explanatory variable; Model 2 is the model for individual-level indicators; Model 3is the full model for individual- and institutional-level indicators; OR, Odds ratios; CI, Confidence interval; PCSP, Per capita service population; HRMP, Human resource management practice; Ref, Reference category; ICC Intra-Class Correlation, LR Test, likelihood ratio test, AIC Akaike’s Information Criterion; ^***^p < 0.01; ^**^p < 0.05; ^*^p < 0.1.

The result of the fixed effects showed that in the individual-level indicators, the decrease in turnover intention was influenced by education and professional title, whereas age, HRMP, and job satisfaction influenced its increase. Regarding education, PMS with a bachelor’s degree or above were 2.40 times (95% CI: 1.56–3.71, *p* < 0.05) more willing to choose to leave compared to those with technical secondary school. Regarding the professional title, the odds of turnover intention increased 51% (95% CI, 1.07–2.12, *p* < 0.05) for PHM with middle titles compared to those with the junior title. Regarding age, the odds of turnover intentions decreased to 33% (95% CI, 0.21–0.51, *p* < 0.01) in aged 41–50 years, and to 23% (95% CI, 0.14–0.38, *p* < 0.01) in more than aged 50 years compared with those in aged 30 years or less. Compared with lower HRMP and lower job satisfaction, the odds of turnover intention decreased to 26% (95% CI, 0.20–0.34, *p* < 0.01) in PMS with high HRMP and declined to 30% (95% CI, 0.21–0.41, *p* < 0.01) in PMS with high job satisfaction.

In institutional-level indicators, the odds of turnover intention increased by 37% (95% CI: 0.97–1.93, *p* < 0.10) for PMS in institutions with more than 250 PCSP people compared with those in institutions with PCSP 250 people or less. In contrast, the odds of turnover intention decreased to 68% (95% CI: 0.50–0.92, *p* < 0.05) for PMS in institutions with more than 50 beds compared with those in institutions with 50 beds or less.

## 4. Discussion

This study is a cross-sectional survey of PMS in representative rural areas of XPCC that reveals, for the first time, the prevalence of turnover intention among PMS in this area and identifies the high-risk population of turnover intentionand the key factors at the individual level that effectively reduce the probability of turnover intention. Furthermore, new and substantial evidence has shown that the characteristics of primary medical institutions may partially explain the occurrence of turnover intentions.

Our data revealed that 20.5% of medical staff had considered leaving their jobs, which is relatively lowerthanother western regions (23.2–33.2%) (Liu and Mao, 2016; Liu et al., 2019; Deng et al., 2022) and lower than the global prevalence estimate (47%) (Shen et al., 2020). However, this does not imply that this region has a low prevalence. First, unlike the above study, the participants were only from public primary medical institutions held by the government, where PMS at these institutions are less likely to leave their jobs than at other types of institutions (Dong and Zhang, 2016). Notably, this result was still higher than those of some national cross-sectional studies (Sun et al., 2013; Li et al., 2020; Kong et al., 2021) and Guangdong, Shanghai, and other regions (Liu et al., 2017; Ou et al., 2018). Therefore, this indicates that the risk of PMS loss in primary medical institutions funded by the government is relatively high. For regions with low geographic accessibility to PMS, this risk will increase the cost of PMS and the cost of overall health services and reduce the utilization of health services for rural residents (Newman et al., 2014).

Unlike previous studies (Liu et al., 2017; Kao et al., 2018; Mao et al., 2018; Liu et al., 2019; Li et al., 2020; Shen et al., 2020; Li et al., 2021), this study found no strong correlation between salary and PMS turnover intention. The influence of age, education, and professional title on the odds of turnover intention is consistent with expectation (Liu and Mao, 2016; Liu et al., 2019; Chen et al., 2021; Deng et al., 2022) but with variations. This study only detected evidence of low odds in the age group 41–50 years and older, without a significant gap observed in younger age groups. Furthermore, this study adds to the evidence that the odds of turnover intention in PMS with a bachelor’s degree or above were 2.4 times that in those with a technical secondary school degree. Moreover, we observed that the odds of turnover intention were in this region’s group with middle titles rather than in the group with primary titles in previous studies (Liu and Mao, 2016; Liu et al., 2019; Shen et al., 2020; Chen et al., 2021; Deng et al., 2022). The above results illustrate that young, highly educated PMS with accumulated practical experience are high-risk populations for this prevalence. Facing the aging situation of the PMS of the XPCC (Lei et al., 2014), the high-quality and sustainable development of the PMS will be greatly affected.

We observed that 67.6 and 44.3% of the participants had higher evaluations of HRMP and job satisfaction, respectively. In addition, we confirmed that high HRMP and job satisfaction could significantly inhibit the occurence of turnover intention. First, it is noteworthy that studies related to HRMP are currently focused on hospitals (He et al., 2020, 2022); therefore, the results of this study can be used as baseline data for predicting the turnover risk of PMS in primary medical institutions. Second, HRMP includes salary, selection, training, and development. From the failure of salary to predict PMS turnover risk, it can be observed that individuals’ perceptions of HRMP have a stronger early warning effect on turnover risk. Finally, a significant correlation between high job satisfaction and turnover intention has been confirmed by several studies (Lee et al., 2016; Liu and Mao, 2016; Liu et al., 2019; Lei et al., 2020), in which <42% of the participants were satisfied with their jobs, which is significantly lower than the results of the 11 provinces in Western China (Liu et al., 2019). Therefore, these findings are of great significance for adjusting and evaluating talent retention measures in later primary medical institutions.

The key strength of our study is that it is the first to confirm that 8.1% of the occurrence of turnover intention in PMS is attributed to the bed number and serving a population of primary medical institutions. The number of beds in primary medical institutions reduced the occurrence of turnover intention in PMS, while the number of serving a population *per capita* had the opposite effect. The former should be attributed to the fact that primary medical institutions with many beds have a larger scale, relatively strong comprehensive management capacity, and a relatively sound incentive mechanisms (Zhang et al., 2020).The latter reflects the level of human resource allocation and PMS workload. The greater the PCSP, the lower the level of human resource allocation, the heavier the work burden, and the more likely the PMS will consider leaving. Therefore, the government should focus on developing talent in small-scale primary medical institutions and strive to improve the fairness of human resource allocation.

### 4.1. Strengths and limitations

We use the multi-level model to explain the spatial aggregation of individual turnover intention at the institutional level to reduce the influence of the hierarchical structure on the results, thereby making the study results robust. This study firstly revealed the characteristics of PMS turnover intention in the XPCC area, which provided a basis for local governments or regions and countries with similar backgrounds to accurately identify high-risk groups and implement accurate talent retention strategies in rural areas. In addition, evidence of a significant correlation between the scale of primary medical institutions and service population size and individual turnover intention may provide a new way to fully understand the determinants of turnover intention prevalence.

Not to be ignored, this study had some limitations. First, although this study included many individual- and institution-level indicators that affect turnover intention, there are still potential confounding factors, including medical insurance, pension insurance, service radius, and hardware facilities of primary medical institutions. Second, the sample size was limited to XPCC. Therefore, if the research results are extended to the entire region of Xinjiang and other regions, it will be necessary to further expand the sample area.

In conclusion, the geographical accessibility of medical resources in the rural areas of the XPCC is poor, and the loss of a few primary medical workers can reduce the utilization of medical services for rural residents. However, we observed that one in five medical workers working in government-run primary medical institutions considered leaving their jobs. From a personal perspective, although junior medical workers over the age of 40 years have a lower probability of turnover intention, those with an undergraduate degree or above and middle titles are at a higher risk. However, we found that individuals with a higher evaluation of HRMP and job satisfaction could also effectively reduce risk. Therefore, organizations should pay attention to the training, development, and use of primary medical workers and create a good working environment. From the perspective of primary medical institutions, larger institutions can reduce the turnover intention of individuals, while the size of the service population has the opposite effect. Therefore, the Government should focus on resource-disadvantaged primary care institutions and equitable distribution of medical workers.

## Data availability statement

The original contributions presented in the study are included in the article/supplementary material, further inquiries can be directed to the corresponding author.

## Ethics statement

The studies involving human participants were reviewed and approved by Ethics Committee of the First Affiliated Hospital, Shihezi University School of Medicine. The patients/participants provided their written informed consent to participate in this study.

## Author contributions

TL conceived and designed the experiments. CT provides project administration and resources. TL, CT, and YL conducted the surveys and collected data. YeL, YuL, and WG analyzed and interpreted the data and wrote the first draft of the article. CT critically revised the article for important intellectual content. All authors contributed to the article, reviewed the manuscript, and approved the submitted version.

## Funding

This study was supported by the National Natural Science Foundation of China (grant number: 72064033).

## Conflict of interest

The authors declare that the research was conducted in the absence of any commercial or financial relationships that could be construed as potential conflicts of interest.

## Publisher’s note

All claims expressed in this article are solely those of the authors and do not necessarily represent those of their affiliated organizations, or those of the publisher, the editors and the reviewers. Any product that may be evaluated in this article, or claim that may be made by its manufacturer, is not guaranteed or endorsed by the publisher.

## Abbreviations

PMS: Primary Medical Staff
XPCC: Xinjiang Production and Construction Corps
HRMP: Human Resource Management Practice
PCSP: *Per capita* Served Population
KMO: KaiserMeyerOlkin
